# StyHighNet: Semi-Supervised Learning Height Estimation from a Single Aerial Image via Unified Style Transferring

**DOI:** 10.3390/s21072272

**Published:** 2021-03-24

**Authors:** Qian Gao, Xukun Shen

**Affiliations:** 1 State Key Laboratory of Virtual Reality Technology and Systems, Beihang University, Beijing 100191, China; xkshen@buaa.edu.cn; 2 School of New Media Art and Design, Beihang University, Beijing 100191, China

**Keywords:** height estimation, semi-supervised learning, style transfer, convolutional neural network, domain adaptation

## Abstract

Recovering height information from a single aerial image is a key problem in the fields of computer vision and remote sensing. At present, supervised learning methods have achieved impressive results, but, due to domain bias, the trained model cannot be directly applied to a new scene. In this paper, we propose a novel semi-supervised framework, StyHighNet, for accurately estimating the height of a single aerial image in a new city that requires only a small number of labeled data. The core is to transfer multi-source images to a unified style, making the unlabeled data provide the appearance distribution as additional supervision signals. The framework mainly contains three sub-networks: (1) the style transferring sub-network maps multi-source images into unified style distribution maps (USDMs); (2) the height regression sub-network, with the function of predicting the height maps from USDMs; and (3) the style discrimination sub-network, used to distinguish the sources of USDMs. Among them, the style transferring sub-network shoulders dual responsibilities: On the one hand, it needs to compute USDMs with obvious characteristics, so that the height regression sub-network can accurately estimate the height maps. On the other hand, it is necessary that the USDMs have consistent distribution to confuse the style discrimination sub-network, so as to achieve the goal of domain adaptation. Unlike previous methods, our style distribution function is learned unsupervised, thus it is of greater flexibility and better accuracy. Furthermore, when the style discrimination sub-network is shielded, this framework can also be used for supervised learning. We performed qualitatively and quantitative evaluations on two sets of public data, Vaihingen and Potsdam. Experiments show that the framework achieved superior performance in both supervised and semi-supervised learning modes.

## 1. Introduction

With the development of remote sensing and image acquisition technology, high-resolution aerial images are widely used, e.g., in urban planning, disaster monitoring, emergency management, and so on. If height information could be automatically extracted from aerial images, it would further improve the intelligent level of downstream applications, such as automated city modeling [1,2], augmented reality [3,4], etc. However, it is a technically ill-posed problem to extract height from a single image [5], especially for the scenes with complex structure. Most traditional solutions are based on handcrafted visual features and probabilistic graphical models (PGMs), which rely on strong assumptions about the geometry of the scene, seriously affected by issues of flexibility and stability [6]. In recent years, with the growth of deep learning and the emergence of large-scale datasets, image-to-height mapping can be trained end-to-end. Most of them use an encoder–decoder network structure [7], where the encoder is responsible for extracting multi-scale spatial features, while the decoder gradually up-samples these features to the original size to obtain dense height values. Although excellent performance has been achieved through supervised learning, there are still some problems in practice: (1) Making training labels is expensive. At present, the mainstream method for obtaining depth/height labels is based on LiDAR or multi-view stereo vision (MVS) 3D reconstruction, both of which require complex and expensive pre/post-processing. Therefore, currently available aerial height estimation datasets are limited in number and scale; thus, training on these datasets easily leads to overfitting. (2) Due to historical, climatic, and cultural reasons, the appearances of different cities are significantly different (domain bias [8]), which means models trained in one city cannot be properly applied to other cities.

To solve the above problems, researchers have proposed many solutions. The authors of [9,10,11] generated synthetic datasets. The virtual 3D world is constructed manually or semi-automatically, and then samples and labels are exported automatically or interactively. This kind of data has the advantages of low cost, fast generation, and no noise, which can effectively make up for the shortcomings of the real dataset. However, there exists a problem named domain bias [8] caused by the difference between the real world and the virtual scenes. Zhou et al. [12] proposed the fine-tuning method to deal with the situation where only a small amount of training data available in the new scene, which locks most of the model parameters trained on the original data and then retrains the remaining parameters on the target data. Although the convergence rate is faster, it can easily cause the phenomena of overfitting and catastrophic forgetting [13]. Atapour et al. [14] proposed a domain adapting method. By firstly training a deep model on synthetic data, and then mapping the real data to synthetic data, it easily gives rise to semantic deviation when the appearance difference between two domains is large (such as two cities).

In this paper, we propose a novel semi-supervised deep neural framework, named StyHighNet, that can accurately estimate the height map from a single aerial image that only requires a small count of training labels. The core is to transfer multi-source data (source domain data with a large number of labels, target domain data with a small number of labels, and synthetic data) into a kind of unified style distribution maps, and then infer the height maps from them. This framework contains three sub-networks: (1) the style transferring sub-network (STN), which converts multi-source images into unified style distribution maps (USDMs); (2) the height regression sub-network (HRN), which infers the dense height map from USDMs; and (3) the style discrimination sub-network (SDN), which determines the source type of USDMs. Among them, STN has a dual responsibility: On the one hand, it tries to estimate the height map accurately together with HRN, therefore making USDMs have sufficient characteristics to regress from. On the other hand, it attempts to confuse SDN in an adversarial manner, making the distribution of USDMs similar, to achieve the goal of domain adaptation [15]. Compared with previous work, the differences in our work are as follows: (1) We discriminate the data source in a dense manner using pixel-wise prediction of the probability distribution of source category, which refines the control of style distribution. (2) We use dual networks to estimate the height maps, which has more learning ability to deal with complex scenes. (3) The distribution of USDMs is learned unsupervised, thus it has great flexibility and stability, especially when there are significant differences between the data sources. In addition, StyHighNet can be regarded as a general learning framework because it is compatible with supervised and semi-supervised learning modes. In summary, the contributions of this article are as follows:

1. We propose a novel network framework that can semi-supervised learn height estimation from a single image based on unified style transferring.
2. We generate a small-scale synthetic dataset automatically through city modeling software and a game engine to make up for the lack of real-world data.
3. We design a set of loss functions that enable three sub-networks to work orderly to achieve the goal of semi-supervised learning.

We conducted quantitative and qualitative evaluations on two public datasets of Vaihingen and Potsdam. The experiments showed that our framework outperforms previous methods in both supervised and semi-supervised mode. We also verified the effects of hyperparameters through an ablation study.

## 2. Related Works

In this section, we review the the three most relevant aspects to the method proposed in this paper, namely monocular depth/height estimation, domain adaptation, and style transfer.

### 2.1. Monocular Depth/Height Estimation

The purpose of monocular depth/height estimation is to determine the depth/height value corresponding to each pixel in the image. It is a basic problem in many computer vision tasks and has received extensive attention. Early methods are mainly based on handcrafted features and probabilistic graph models (PGMs). Saxena et al. [16,17] used Markov Random Fields (MRF) and combined local/global features to infer the depth from the monocular image, and introduce super-pixels to achieve neighboring constraints. Comber et al. [18] calculated the height of the building based on the relationship between length of the shadow and the pose of the sun. Qi et al. [19,20] used the information provided by Google Earth to propose CSLR (corner shadow length ratio) to calculate the height of the building. These methods rely on the strong assumption of the input image thus have some limitations in practical applications.

In terms of deep learning, Baig et al. [21] used sparse coding to estimate the depth of the entire scene. The authors of [22,23] used a two-scale network to learn the mapping of RGB images to depth. Since then, there have been multiple improved versions [24,25,26,27]. In the field of remote sensing, several networks for predicting height have been proposed [5,28,29,30,31]. The above methods generally adopt decoder–decoder structures, where the encoder extracts multi-scale features, and the decoder up-samples and combines these features to regress the pixel-wise height. However, due to the lack of high-quality/large-scale training data, these supervised learning methods suffer from the problems of stability and integrity [32]. Recently, Xie et al. [33] proposed a self-supervised learning method, Deep3D network, to predict the depth map from stereo images without training labels, which reconstructs a virtual right image with a predicted depth map and known camera translation, and the consistency relative to the left image is utilized as mainly leaning signal. Godard et al. [34] used bilinear difference and left-right consistency cross-validation to obtain higher accuracy. Although such methods achieve superior quality to the supervised version, the stereo image pairs require strict synchronization and calibration that still limit the training data. Zhou et al. [35] simultaneously estimated the depth map and ego-motion of the adjacent frame within a monocular video, which further reduced the threshold of training data, but the dynamic objects in the scene violate the assumption of rigid transformation, leading to a fuzzy and incomplete result. Subsequent work made improvements in this area by off-line masking [36,37,38], optical-flow [39] or on-line masking [32,40,41]. The authors of [42,43] proposed a semi-supervised learning depth estimation method, which combines the use of LiDAR labels and the consistency of the novel view of adjacent frames to ensure the correct prediction. Sex et al. [44] proposed a method that semi-supervised learns the depth estimation of a single image through the relationship between semantic labels and geometric information. Although the above methods achieved high fidelity on the training data, the situation of cross-domain is not considered. Moreover, in the field of remote sensing, isolated images without spatiotemporal adjacent frames are the mainstream data format. Therefore, we take advantage of both height labels and unified style distribution as learning signals to achieve accuracy and domain adaptability simultaneously.

### 2.2. Domain Adaptation

Due to the lack of comprehensive training datasets for depth/height estimation, synthetic datasets [9,10,11] were generated as a complement for the real-world datasets through their low cost and perfect pixels. However, the inevitable bias that comes from the virtual modeling and rendering process makes the networks trained on synthetic images cannot directly apply to real-world scenes. Zhou et al. [12] proposed a fine-tuning method that retrains the model on a small count of target data, but it faces the issues of overfitting and catastrophic forgetting [13]. Domain adaptation methods [8,15,45,46,47,48,49] minimize the difference between the source data and the target data by a pre-trained model, but they tend to fail when the difference between two data sources is large (e.g., two cities). Here, we learn a unified style distribution unsupervised to avoid the phenomenon of adaptation failure.

### 2.3. Style Transfer

The method of Gatys et al. [50] firstly converts source images to another style via a convolutional neural network. The subsequent methods directly update the pixel value of the output image [51,52,53,54] or learn the specified image style from a large amount of training data [55,56,57,58,59]. Among them, the Gram Matrix is usually used to evaluate the consistency of the distribution. Inspired by this idea, we transfer multi-source images to a unified style distribution and preserve the obvious characteristics at the same time to ensure the robustness of height estimation.

## 3. Method

In the following subsection, we introduce the implementation details of the proposed framework, namely pipeline overview, running mechanism, and loss functions.

### 3.1. Pipeline Overview

**Figure 1:**
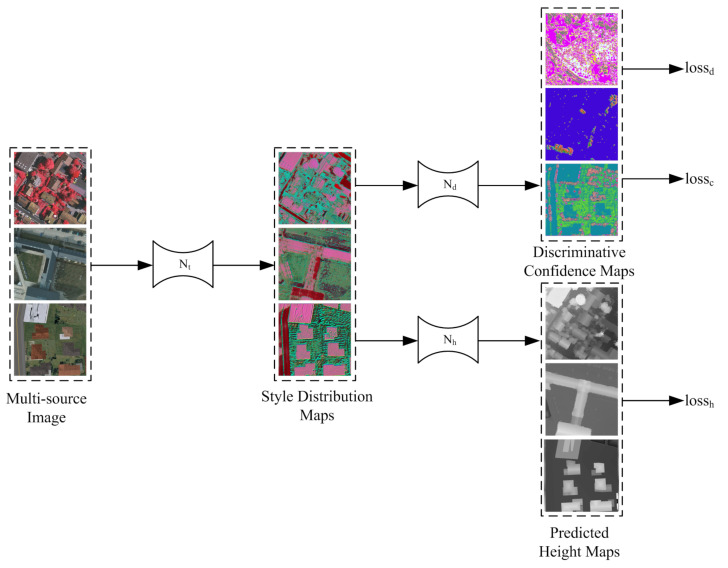
Pipeline overview. The framework contains three sub-networks: style transferring sub-network ($N_{t}$), height regression sub-network ($N_{h}$), and style discrimination sub-network ($N_{d}$). They work together to complete the task of semi-supervised height regression.

The framework is composed of three sub-networks: (1) The style transferring network $N_{t}$, which converts the original image $X_{⋆}\in R^{H\times W\times 3}$ from multiple sources into the style distribution maps $T_{⋆}\in R^{H\times W\times C_{t}}$, where ⋆∈{sup,sem,syn} represents the three types of input images, sup represents to the real data with a large number of labels, sem means the real data with a small number of labels, and syn refers to the synthetic data; (2) the height regression network $N_{h}$, which regresses the height maps $Y_{⋆}\in R^{H\times W\times 1}$ from $T_{⋆}$; and (3) the style discrimination network $N_{d}$, with inputs $T_{⋆}$ and outputs $D_{⋆}\in R^{H\times W\times 3}$, which represent the probability distribution of source category of $T_{⋆}$. These three sub-networks are coupled together to achieve the goal of height estimation and domain adaptation through three loss functions ($loss_{h},loss_{d},loss_{c}$), as shown in Figure 1.

### 3.2. Implementation Mechanism of StyHighNet

In our pipeline, there are two workflows trained simultaneously: one is supervised learning of height regression (including $N_{t}$ and $N_{h}$) and the other is unsupervised learning of unified style distribution (including $N_{t}$, and $N_{d}$). It can see that $N_{t}$ undertakes dual tasks in these two workflows to achieve the purpose of semi-supervised learning.

#### 3.2.1. Supervised Height Regression

Unlike the previous supervised method [29,60], our height estimation adopts a dual-network serial inference strategy. The style transferring network $N_{t}$ converts the multi-source images $X=\{X_{⋆}|⋆=sup,sem,syn\}$ into the style distribution maps *T* and regresses them by height regression network $N_{h}$ to the corresponding height maps *Y*. There are three types of sources of input data for $N_{t}$, each of them playing a different role: (1) real data with many labels $X_{sup}$ are the main force of supervised learning and are the source domain in terms of domain adaptation; (2) real data with few labels $X_{sem}$, which, although the number is not large, provide the key guidance to style distribution and are the target domain in terms of domain adaptation; and (3) synthetic data $X_{syn}$ are used as a complement to $X_{sup}$ because of their low cost and perfect pixels.

**Figure 2:**
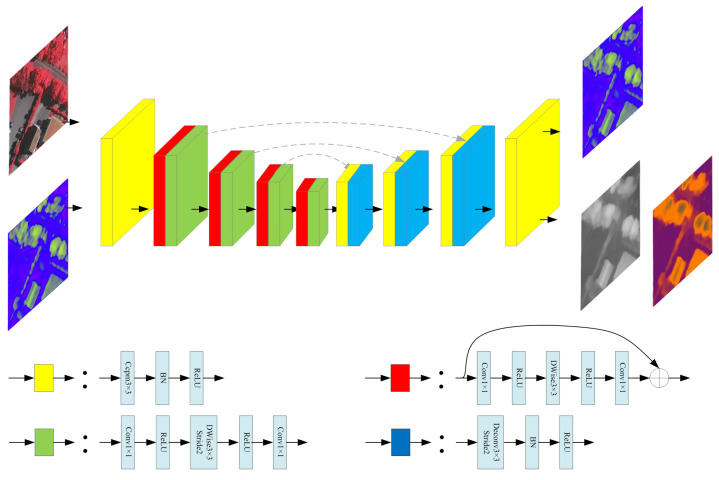
Sub-network architecture. The three sub-networks use the same network structure, but according to different specific tasks, the input and output data are different.

We employ a popular encoder–decoder structure [7] for both $N_{t}$ and $N_{h}$. The encoder adopts the MobileNetV2 architecture [61] to improve the computational efficiency. The decoder uses deconvolution as the up-sampling function. The feature maps with the same size in the encoder and decoder are skipped and connected to preserve the geometric details. The input and output sizes of the two networks are the same, and the number of channels of *T* is set to 3 for the convenience of visualization and analysis. The output activation functions of $N_{t}$ and $N_{h}$ are both sigmoid. The specific network structure is shown in Figure 2.

#### 3.2.2. Unsupervised Style Transferring

The task of unsupervised style transferring is jointly completed by the style transfer network $N_{t}$ and the style discrimination network $N_{d}$. Their relationship is similar to that of generator and discriminator in Generative Adversarial Networks (GANs) [62]. $N_{d}$ is used to judge (classify) the source category of the $T=\{T_{*}|*=sup,sem,syn\}$, output number of channels is the number of source categories (here is 3), and the activation function is softmax to form the probability distribution of classification. $N_{t}$ tries to confuse $N_{d}$, which makes the distribution of *T* from multi-source images as similar as possible, to achieve the purpose of domain adaptation. The unified style distribution is not known in advance; it is learned unsupervised and tends to be stable during the adversarial process. However, two points are different from the classic generative confrontation network [62]: (1) The $N_{d}$ network performs the classification task for each pixel, rather than summarizes them into a scalar to distinguish, making control and analysis further improved. (2) Our style distribution maps *T* are derived from the multi-source images *X* instead of a random vector. We use the same network structure for $N_{d}$, as shown in Figure 2.

#### 3.2.3. Semi-Supervised Learning

During the training process, the two workflows mentioned above are carried out at the same time. It is clear that the style transfer network $N_{t}$ shoulders dual tasks simultaneously: On the one hand, it supervised learns a style transferring function together with height estimation network $N_{h}$ to recover the height map from multi-source images. The characteristics of the style distribution map *T* need to be obvious to achieve the goal of accurate height regression. On the other hand, it cooperates with the style discrimination network $N_{d}$ in an adversarial manner to make the *T* as similar as possible to achieve domain adaptation. Therefore, the images without labels can also contribute their supervising signals on style distribution. Note that the labeled data only enter the height regression workflow, while all of the data enter the style transferring workflow, which forms a semi-supervised learning mechanism. In the training phase, these two workflows are performed cooperatively in parallel.

### 3.3. Loss Functions

Style transferring sub-network $N_{t}$ and height regression sub-network participate in supervised learning to recover the height map from multi-source images. The binary-cross-entropy (BCE) $loss_{h}$ is used to optimize the parameters in these two sub-networks, namely

(1) $$loss_{h}=-\frac{1}{N}\sum_{i}\sum_{⋆}\left[{\hat{Y}}_{⋆,i}\log\left(Y_{⋆,i}\right)+\left(1-{\hat{Y}}_{⋆,i}\right)\log\left(1-Y_{⋆,i}\right)\right],⋆\in \left\{sup,sem,syn\right\}$$

where

(2) $$Y_{*}=F\left(N_{h},F\left(N_{t},X_{⋆}\right)\right)$$

is the predicted height map, F(·,·) denotes the network mapping function, *N* is the number of all pixels, *i* is the pixel index, and $\hat{Y_{⋆}}$ is the corresponding height labels.

In the optimization process, the output style distribution maps $T_{⋆}=F\left(M_{t},X_{⋆}\right)$ from the style transferring sub-network $N_{t}$ are originally unconstrained, thus images from the different data source may have different styles, which leads to domain bias. To this end, we introduce a style discrimination network $N_{d}$ to unify the style distribution, where two losses are involved ($loss_{d}$ and $loss_{c}$). $loss_{d}$ is to evaluate the effect of classification for data categories, achieved by the cross-entropy function [63] similar to the tasks of semantic segmentation [64]. In contrast, $loss_{c}$ aims at confusing the style discrimination network $N_{d}$, making the *T*s from three data categories as similar as possible. They are defined as follows:

(3) $$loss_{d}=\frac{1}{N}\sum_{i}\left[-log\left(D_{m,i}^{m}\right)\right],$$

(4) $$loss_{c}=\frac{1}{N}\sum_{i}\left[-log\left(D_{m,i}^{0}\right)\right]$$

where $D\in R^{H,W,3}$ is the output of style discriminant sub-network $N_{d}$, which is normalized by a softmax activation. $D_{m,i}^{l}$ is the discriminant probability inferred from *m*th data source category at the position of pixel *i* and in the *m*th channel, where m,l∈{0,1,2}.

If we treat *D* as an RGB image, the style discriminant network $N_{d}$ tries to output three pure color images for three data categories: red for $T_{sup}$, green for $T_{sem}$ and blue for $T_{syn}$. In Equation (4), we set the target category always be 0, as Xsup has the most learning signals that can avoid the phenomenon of excessive smoothness. Other style distribution maps ($T_{sem}$ and $T_{syn}$ are constrained to be closed with $T_{sup}$ to accomplish the task of domain adaptation.

The height regression sub-network $N_{h}$ and style discriminant sub-network $N_{d}$ are optimized by $loss_{h}$ and $loss_{d}$, respectively, as they are both independent modules. However, style transferring sub-network $N_{t}$ is a dual-task module, so it has a combined loss function:

(5) $$loss_{t}=loss_{h}+\lambda loss_{c}.$$

where the coefficient λ is a fusing weight, and set to be 0.1 in practice.

## 4. Experiment

To verify the performance of the ThickSeg, we built a synthetic dataset and made a qualitative and quantitative evaluation on two open datasets of Vaihengen and Potsdam. We also performed an ablation study to observe the effects of hyper-parameters.

### 4.1. Datasets

Vaihingen dataset includes 33 regions of different sizes, each of them containing a top view taken from the mosaic and the corresponding height map. The ground sampling interval of the two types of images is all 9 cm. The height maps are generated by Trimble INPHO 5.3 software, and the top views are stitched by Trimble INPHO OthoVista. To avoid data loss, these 33 areas are sliced in the center part of the reconstructed scene, where interpolation is used to remove missing data.

Potsdam dataset contains 38 areas with the same size, where top views and height maps are both taken from the mosaic with 5 cm sampling spacing. The top view images are in TIFF format and have different channel combinations: (1) IRRG with three channels (IR-RG); (2) RGB with three channels (RGB); and (3) RGBIR with four channels (RGB-IR). Users can choose the appropriate channel mode, and here we use RGB mode. The height maps are also in TIFF format but with one channel, and are coded as a 32 bit floating point in meters.

**Figure 3:**
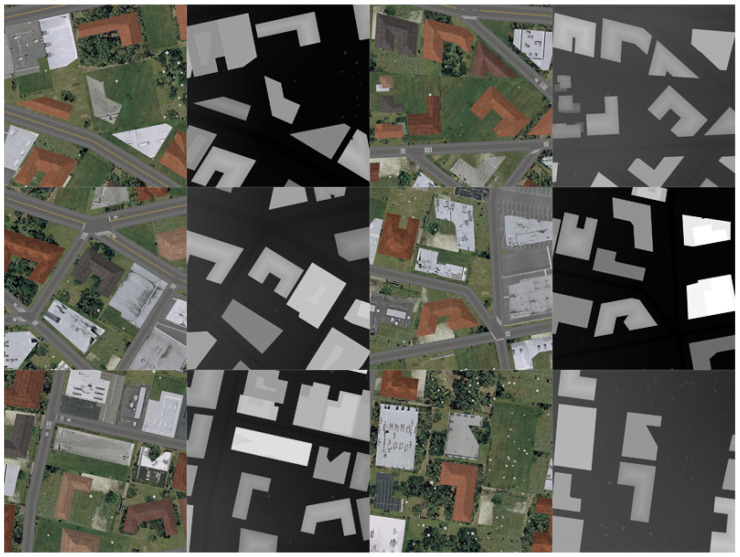
Synthetic dataset. The first and third columns are rendered color images and the second and fourth columns are the corresponding height maps where the intensity is proportional to their height values.

Synthetic dataset, similar to the one in [65], is generated automatically by modeling software and a game engine. Objects are randomly distributed in the virtual city, including roads, buildings, trees, lawns, etc. The 3D models are imported into the game engine through obj format, containing shapes, materials, and textures. The color maps and height maps are sampled and rendered at random positions, both in the format of PNG. Some examples are given in Figure 3.

### 4.2. Implementation Details

We implemented the proposed network using the open deep learning framework PyTorch [66]. For training, we used Adam optimizer [67] with $lr=10^{-4}$, $\beta_{1}=0.9$, $\beta_{2}=0.999$, and $\epsilon =10^{-8}$. The learning rate was scheduled via exponential decay with d=0.96. The total number of epochs was set to 50 with batch size 32 on a workstation equipped with four NVIDIA 1080ti GPUs for all experiments in this work.

All three sub-networks adopted U-Net architecture [7] with MobileNet-v2 [61] encoder and de-convolutional decoder. All outputs of sub-networks were filtered by Sigmoid activation for normalization, except for the style discriminant sub-network. for which the output was activated by softmax function for pixel-wise classification. Two workflows of height regression and style transferring were parallel on the macro-level and serial on the micro-level, which means they were trained in turn on each batch.

To avoid overfitting, we augmented images before input to the network using random rotation in the range of [−π,+π] as well as random contrast, brightness, and color adjustment in a range of [0.8, 1.2], with 50% of chance. The images were also randomly cropped to 512×512 and 1024×1024 for training and testing, respectively. Training data and testing data were randomly split according to the radio of 6:4. All test results shown in this section were obtained from the average of five independent experiments. For Potsdam dataset, all original data were down-sampled by radio 2 to expand the sampling distance from 5 to 10 cm.

We used the same numerical metrics as in [29,60] to evaluate the quality of height regression, root-mean-square error (RMSE) and the zero-mean normalized cross-correlation (ZNCC), which are defined as:

(6) $$RSME=\sqrt{\frac{1}{N}\sum_{i}^{N}{\left(x_{i}-y_{i}\right)}^{2}}$$

(7) $$ZNCC=\frac{1}{N}\sum_{i}^{N}\frac{1}{\sigma^{x}\sigma^{y}}\left(x_{i}-\mu_{x}\right)\left(y_{i}-\mu_{y}\right).$$

where *x* and *y* denote output and ground truth, respectively, with *n* pixels. $\mu_{x}$ and $\mu_{y}$ are the mean values of *x* and *y*, while $\sigma_{x}$ and $\sigma_{y}$ are the standard deviations of *x* and *y*.

### 4.3. Supervised Mode

**Figure 4:**
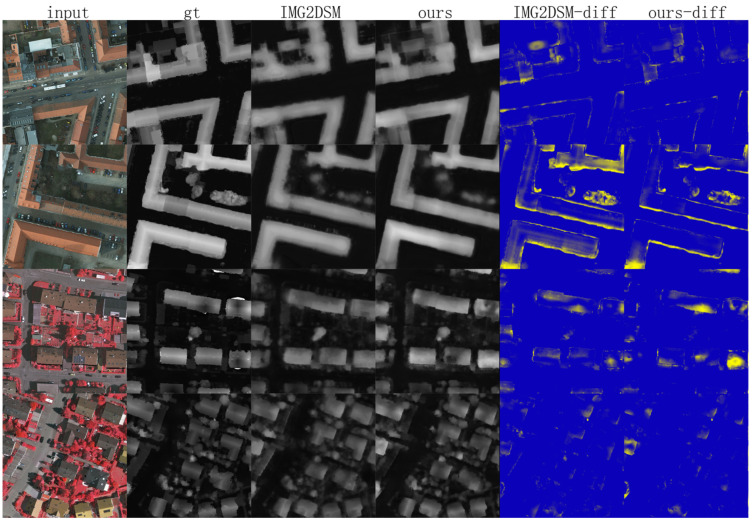
Two examples from the Potsdam dataset (top two rows) and two examples from the Vaihingen dataset (bottom two rows). From left to right, we show the input images, ground truth, predicted height maps of IMG2DSM (our implementation), predicted height maps of our method, height difference maps using IMG2DSM, and height difference maps of our result, respectively.

Our framework supports supervised learning by simply neglecting the style discriminant sub-network. In this learning mode, two datasets (Vaihingen and Potsdam) were trained separately as only one source data ($X_{sup}$) is needed. $X_{sup}$ was firstly inputted into style transferring sub-network $N_{t}$ to get a style distribution map *T*, and then, *T* was fed to the height regression sub-network $N_{h}$ to regress the height maps where the only $loss_{h}$ was minimized to optimize both sub-networks jointly. As shown in Table 1, measurements of RMSE and ZNN were improved by 2% and 3%, respectively, compared to state-of-the-art work [60]. Visualized results are shown in Figure 4 and compared with IMG2DSM [29], where can be inferred that our result is sharper than that of IMG2DSM [29].

Although StyHighNet needs two cascaded sub-networks to predict the height maps, it still achieves a high level of time and space efficiency. All sub-networks in StyHighNet were implemented by a lightweight structure of MobileNetV2 [61], which only contains 12M parameters parameters and predicts a 1024×1024 image in just 50 ms.

### 4.4. Semi-Supervised Mode

#### 4.4.1. Inner-Domain Semi-Supervised Learning

**Figure 5:**
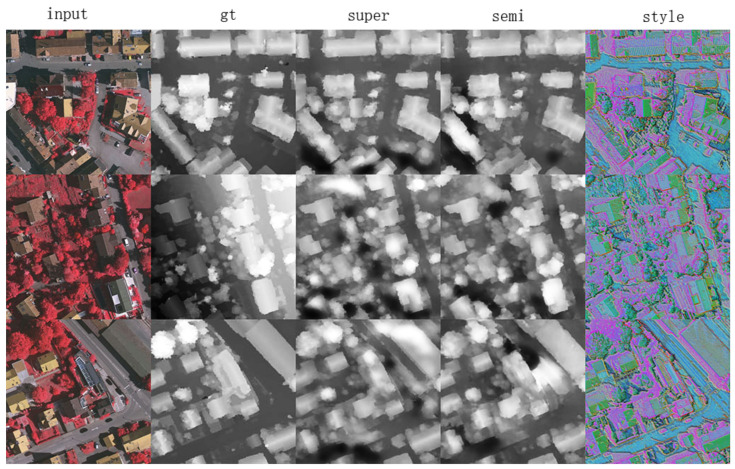
Three examples from the Vaihingen dataset in inner-domain semi-supervised learning mode. The columns from left to right correspond to the test images, ground truth, supervised learning results, semi-supervised learning results, and corresponding style distribution maps.

In the inner-domain semi-supervised mode, the training data in each dataset were further split into two parts: the images with or without labels to simulate circumstance where many images exist of one city but few of them are labeled due to the cost of annotation. We performed the experiments on two datasets (Vaihingen and Potsdam) separately; the ratio of the labeled images were set as 20%, 50%, and 80%. In this mode, three sub-networks ($N_{t}$, $N_{h}$, and $N_{d}$) were all trained as described in Section 3.2.3, and three loss functions ($loss_{t}$, $loss_{h}$, and $loss_{d}$) were all involved, with the fusing weight λ in Equation (5) set to 0.1. We compared the results to those of the supervised mode introduced in the last section, as shown in Table 2, which only used labeled images for learning. The results of the semi-supervised mode are superior to those of the supervised mode because extra data (unlabeled data) were used to constrain the style distribution maps, thus avoiding overfitting. The visualization results are shown in Figure 5.

#### 4.4.2. Inter-Domain Semi-Supervised Learning

**Figure 6:**
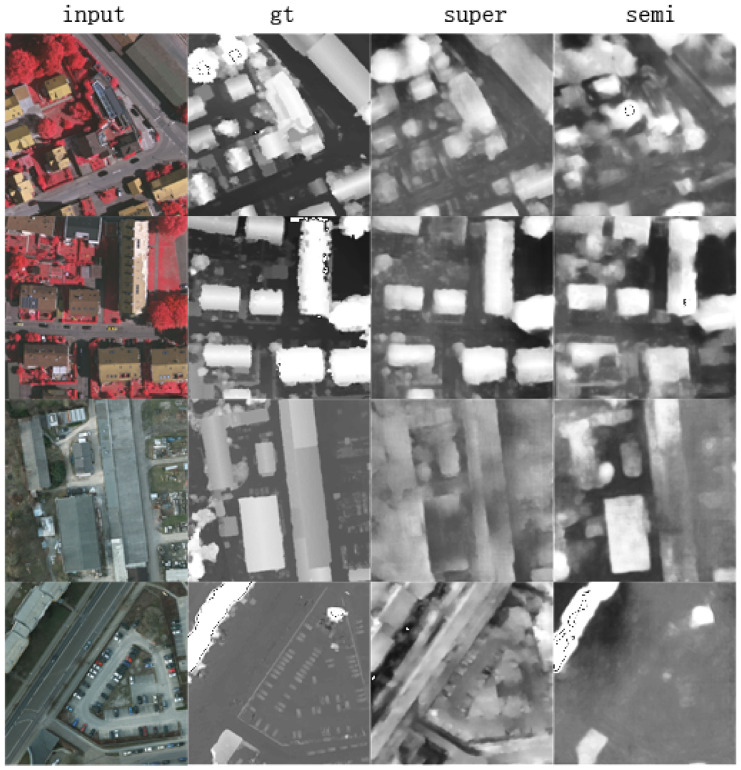
Inter-domain semi-supervised visualization. From left to right are input images, ground truths, supervised learning predictions, and semi-supervised learning predictions.

The inter-domain semi-supervised learning mode was also designed for the circumstance of lack of labeled images. In contrast to the inner-domain mode, this mode focuses on the problem of domain bias, in which the model trained in one city has difficulty being applied in another city, which is very common in practice. We used all the training data from one city with a small percentage of labeled data (20%) from another city as the supervised signals of height regression, and the remaining unlabeled data were used for unsupervised learning of style distribution. We used the same parameters as in the previous section to train and test the model and compared the results to the supervised learning method, fine-tuning [12], and with or without synthetic data, as shown in Table 3. The inter-domain configuration achieves the best result, as the unlabeled data contributed to constrain and unify the style distribution. Furthermore, the use of synthetic data enhanced the performance significantly. The visualized results are shown in Figure 6.

### 4.5. Ablation Study

We examined two super parameters: the number of channels of style map $n_{t}$ and the loss function of height regression $loos_{h}$. For $n_{t}$, we chose 1, 3, and 5, as shown in Table 4. We observed that overall performance improves with the increase of $n_{t}$ since a thicker style map carries more features for height regression. However, the effect is not obvious when $n_{t}$ increases from 3 to 5 as a three-channel style map can already describe the latent information for this task. For $loss_{h}$, we compared it with the root-mean-square-error (RMSE) loss. We found that the binary-cross-entropy (BCE) loss used in this work outperforms the version with RMSE, as BCE loss tends to form a sharper effect which is more suitable for building-like objects.

## 5. Conclusions

In this paper, we propose a novel framework, named StyHighNet, for semi-supervised learning height estimation from a single aerial image. StyHighNet consists of three sub-networks with the same structure for style transferring, height regression, and style discrimination, respectively. These sub-networks are optimized orderly within two workflows: supervised height regression and unsupervised style transferring. We created a synthetic dataset and performed qualitative and quantitative analysis on two public datasets of Vaihingen and Potsdam. The experiments indicated that StyHighNet is superior in both supervised learning mode and semi-supervised learning mode. Especially in inter-domain semi-supervised learning mode, StyHighNet effectively solves the problem of domain bias in the case of lack of labels. The super parameter of number channels in the style distribution map and the choice of loss function for height regression were analyzed in the ablation study.

## Figures and Tables

**Table 1:** Comparison height estimation results in supervised learning mode with the previous works of IMG2DSM [29] and MPFupsion [60]. Best results in each category are in bold.

	Vaihingen		Potsdam	
Method	RMSE	ZNCC	RMSE	ZNCC
IMG2DSM [29]	2.58	0.759	3.89	0.718
MPFusion [60]	2.45	0.847	3.90	0.821
Ours	**2.40**	**0.872**	**3.83**	**0.845**

**Table 2:** Inner-domain semi-supervised learning results compared with the supervised learning mode on datasets of Vaihingen and Potsdam. Best results in each category are in bold.

Dataset	Method	20%		50%		80%	
		RSME	ZNCC	RSME	ZNCC	RSME	ZNCC
Vaihingen	super	3.830	0.673	3.033	0.776	2.887	0.805
	semi	**3.533**	**0.713**	**2.894**	**0.796**	**2.794**	**0.813**
Potdam	super	4.753	0.667	4.256	0.831	4.035	0.846
	semi	**4.364**	**0.732**	**4.083**	**0.834**	**3.977**	**0.847**

**Table 3:** Comparison of inter-domain semi-supervised learning to supervised learning, fine-tuning [12], and with or without synthetic data, on Vaihingen and Potsdam datasets. Best results in each category are in bold.

Method	Vaihingen		Potsdam	
	RMSE	ZNCC	RMSE	ZNCC
super	4.369	0.506	4.453	0.683
fine-tune [12]	4.536	0.406	5.332	0.323
semi	4.011	0.500	4.557	0.669
semi-syn	**3.491**	**0.681**	**4.245**	**0.712**

**Table 4:** Ablation study. Comparison of the number of channels of style distribution maps and the choice of the height regression function. Best results in each category are in bold.

${\mathrm{loss}}_{h}$	$n_{t}$	Vaihingen		Potsdam	
		RMSE	ZNCC	RMSE	ZNCC
RSME	1	4.212	0.435	4.767	0.425
	3	3.520	0.657	4.256	**0.688**
	5	**3.493**	**0.662**	**4.253**	0.685
BCE	1	4.021	0.455	4.762	0.433
	3	3.491	**0.681**	4.245	**0.712**
	5	**3.482**	0.677	**4.242**	0.708

## Data Availability

Not applicable.
